# Label-Free Exosomal Detection and Classification in Rapid Discriminating Different Cancer Types Based on Specific Raman Phenotypes and Multivariate Statistical Analysis

**DOI:** 10.3390/molecules24162947

**Published:** 2019-08-14

**Authors:** Ping Zhang, Limin Wang, Yaping Fang, Dawei Zheng, Taifeng Lin, Huiqin Wang

**Affiliations:** College of Life Science and Bioengineering, Beijing University of Technology, Beijing 100124, China

**Keywords:** exosomes, biomarker, surface enhanced Raman scattering, cancer, Raman phenotype, rapid detection, label-free

## Abstract

Exosomes contain different functional bimolecular characteristics related to physiological or pathological processes and are now recognized as new biomarkers in different human cancers. Rapid detection and classification of cancer-related exosomes might be helpful in the rapid screening of patients that may have cancer. Here, we report a surface enhanced Raman scattering technology for rapid and label-free exosomal detection (Exo-SERS) to aid in the discrimination of different cancer cells based on specific Raman phenotypes and multivariate statistical analysis. The results demonstrated that exosomes derived from both tumor cells and normal cells exhibit special, unique Raman phenotypes. Using the Exo-SERS method, the cancer cells were accurately discriminated from normal cells, and subtle molecular changes between the different cell types could be detected with high sensitive. This research provides a rapid, label-free and non-destructive manner for detecting and discriminating between cancer types.

## 1. Introduction

Exosomes are nano-sized phospholipid bilayer-enclosed vesicles that are secreted by all cells into the extracellular milieu [1]. Released exosomes contain cell-specific proteins, membrane lipids, mRNA, DNA and microRNA that can perform versatile roles in normal or diseased processes [2,3,4]. Growing evidence indicates that exosomes are plentiful in the body fluids of cancer patient and can transfer certain oncogenic substances that regulate the tumorigenesis and progression [5]. Of note, the exosomes produced by highly metastatic cells developed much more metastatic tissue. Lyden has proposed that exosomes released by tumors are considered malignant messengers that prime distant organs for metastasis and recruit bone marrow cells to assist in this process. Organotropic metastasis was related to the protein expression patterns in exosomes, in which exosomal integrins α6β4 and α6β1 were associated with lung metastasis and integrin αvβ5 was linked to liver metastasis [6]. In 2015, Melo et al. reported that glyican-1 (GPC1), a cell surface proteoglycan, was specifically enriched in cancer cell-derived exosomes and could distinguish pancreatic cancer from non-cancer subjects with absolute specificity and sensitivity. GPC1 levels in the circulating exosomes correlated with tumor burden and the survival of pre- and post-surgical patients. This was the first report showing that circulating exosomes may serve as a potential non-invasive diagnostic and screening tool for the detection of the early stages of pancreatic cancers, thus facilitating surgical therapy [7]. Aside from proteins, exosome-borne RNAs are another important and abundant cargo. It has been reported that elevated amount of serum exosomal miRNA-23a may have potential clinical relevance and prognostic value in lung cancer patients [8]. In view of the stable presence of exosomes in human body fluids, they are now increasingly recognized as important vehicles and vital circulating biomarkers for the detection of cancer. Therefore, the rapid detection of tumor-related exosomes has contributed to the development of non-invasive techniques for cancer monitoring.

To date, the rapid detection of exosomes is still a challenging task. Current methods include electron microscopy (EM), dynamic light scattering (DLS), nanoparticle tracking analysis (NTA), resistive pulse sensing (RPS), western blot, flow cytometry, and mass spectrometry [9,10,11]. NTA, DLS and RPS can provide information on both particle sizes and distribution but are insufficient for the identification of particle type. EM is the gold standard for imaging exosomes because it can simultaneously image vesicle morphology and size. However, the expense of the equipment needed to perform EM is a major limitation. Western blotting is a destructive and indirect assay that is based on the expression of characteristic proteins in exosomes, such as the tetraspanin (CD9, CD63 and CD81), heat-shock proteins (Hsp60, Hsp70 and Hsp90), and proteins involved in multivesicular body biogenesis (TSG101 and ALIX). Western blotting alone cannot qualify and quantify exosomes. Flow cytometry not only enables analysis of particle size, but it can also identify the source and sub-population of exosomes via cell fluorescence labeling. However, standard flow cytometry is not suitable for detecting the cell particles less than 300 nm. LC-MS/MS is the preferred method for studying exosomal proteomics and nucleomics, but the results are often skewed by the complexity of biological components. Therefore, there is still a need to develop new methods for rapid detection of exosomes to aid in cancer identification using scalpel-free biopsies.

Surface enhanced Raman scattering (SERS) technology provides a promising perspective on fast, sensitive, nondestructive, and label free detection. The enhanced Raman signals belong to test samples absorbed on the rough metal surfaces used, which are typically the Ag or Au nanoparticles in the colloid. As a laser beam penetrates through the suspension colloid, the collected SERS signals are a reflection of the whole detecting systems; therefore, SERS may be used to gain the whole fingerprint spectrum of the analytes. Theoretically, exosomes from a variety of different origins have specific functional bio-macromolecules. This means that exosomes from variety of origins have specific genotypes and molecular phenotypes, which in turn can reflect different SERS phenotypes. Therefore, combining SERS technology with exosomal detection (Exo-SERS) to discriminate cell origin is likely feasible according to specific Raman phenotypes and multivariate statistical analysis. Stremersch et al. reported, for the first time, the capability of applying SERS for distinguishing melanoma cell B16F10-derived exosome-like vesicles from red blood cells [12]. Subsequently, the similar studies using SERS method to rapid detect extracellular vesicles and exosomes as the biomarkers to discriminate cancer have been reported recently, including exosomes collected from human lung cancer cells [13], prostate cancer cells [14], breast cancer cells [15], and pancreatic cancer cell lines [16]. Furthermore, labeled SERS detection based on immune-SERS tags was developed to examine the potential of exosomal proteins and RNAs for cancer diagnosis in clinical analysis [17,18,19]. However, the labeled methods relay closely on the synthesis of enhanced substrates, and these processes are complex, tedious, time-consuming, and have low stability, thus restricting application for clinical uses, especially for point-of-care testing.

Based on the above studies, it is clear that the cancerous exosomes can be easily distinguished with normal exosomes due to the presence of various bio-functional components of the SERS technology. However, as one of the potential biomarkers in liquid biopsy techniques, the question of whether different types of cancerous exosomes can be rapidly discriminated still needs to be addressed. Therefore, the overall goal of this work is to rapid discriminate exosomes collected from different cancer types in a label-free manner which has the potential of facilitating the nondestructive cancer diagnosis. The exosomes collected from eight cell lines (human esophageal cancer cells EC109, EC9706 and Kyse150, cancerous breast epithelial cells M231 and MCF7, hepatoma cells HepG2, human normal hepatocyte cells L02 and human nontumorigenic breast epithelial cells MCF-10A) were rapidly detected and classified based on the label-free SERS method. Because the Raman signals can be collected in several seconds, the Raman fingerprints could reliably reflect the different combinations of cargoes in vesicles. It is therefore easy to compare and distinguish the Raman phenotypes of cancerous exosomes from those of normal cells. In conjunction, a multivariate statistical analysis was subsequently applied to further discriminate between the different types of exosomes. The characterization of various types of exosomes would be benefit for nondestructively monitoring maternal neoplastic tissue types through liquid biopsy. Apart from diagnostic applications, this method is potentially useful for deepening insight into the molecular composition/diversity of vesicles secreted by different cancer types. Consequently, these results will pave the way for the development of liquid biopsy for cancer detection and diagnosis.

## 2. Results and Discussion

### 2.1. Exosomes Isolation and Characterization

**Figure 1:**
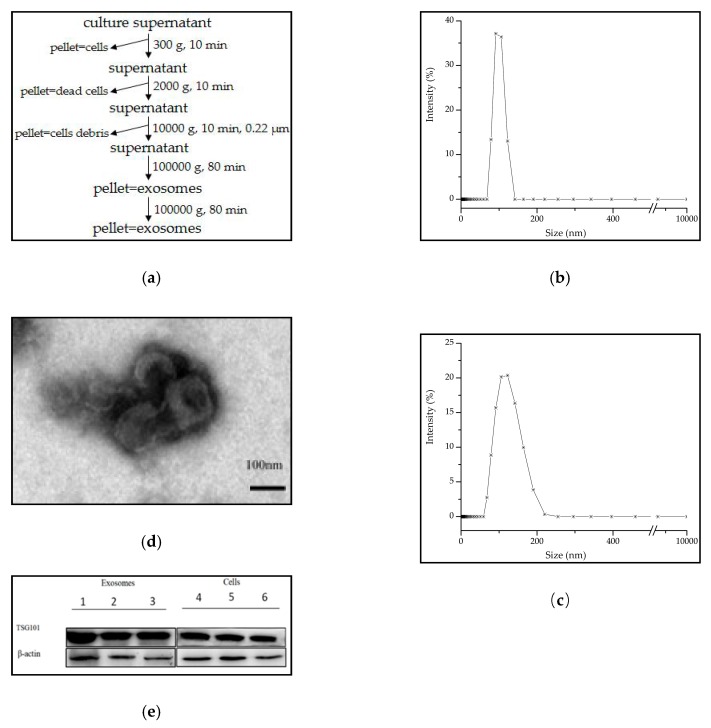
Exosomes isolation and characterization. (**a**) The isolation method of the culture cell-derived exosomes; (**b**) DLS result showed the size of MCF7 cell-derived exosomes about 100 nm in mode; (**c**) and EC109 cell-derived exosomes about 120 nm; (**d**) TEM displayed the morphology and size of exosomes which were negatively stained; (**e**) Detection of TSG101 expression on cell derived exosomes (1–3) and its original cells (4–6), β-actin as control. Note: 1. MCF7-exosome; 2. M231-exosome; 3. HepG2-exosome; 4. MCF7 cell; 5. M231 cell; 6. HepG2 cell.

Exosomes were isolated via differential centrifugation combined with ultracentrifugation of the supernatants of eight cultured cell lines that contained several types of shed membrane fragments and vesicles (Figure 1a).

Therefore, it was critical to ensure that the extracted exosomes were purified vesicles with no or very little contaminating material before performing detection analysis. To avoid the presence of large vehicles, differential centrifugation methods were used in addition to a 0.22 μm micron filter before performing ultracentrifugation to ensure that the obtained exosomes were as pure as possible. Traditional methods were applied to identify the extracted exosomes. The dynamic light scattering (DLS) data showed that the particle size distribution of exosomes collected from MCF7 cells was between 60 nm and 130 nm, with an average diameter of 110 ± 17 nm (Figure 1b). The other cell-derived exosomes were also in this range and did not exceed 150 nm (Figure 1c). The morphology observed under the transmission electron microscopic (TEM) resembled that of a red blood cell or circular plate. The size ranged from 30 nm to 100 nm which was smaller than the DLS results because the exosomes shrank under drying conditions, making the edge of the vesicles ‘cocked up’ (Figure 1d). According to the TEM results, the collected exosomes were pure and homogenous with little vesicle fragment contamination. The exosomal biomarker TSG101 which is involved in multi-vesicular body biogenesis, was examined by immunoblotting methods. In Figure 1e, the expression of TSG101 in MCF7-, M231- and HepG2 cell-derived exosomes were higher than that in its counterpart cells.

### 2.2. The Raman Signals of Exosomes Were Enhanced by Au Nanoparticles

**Figure 2:**
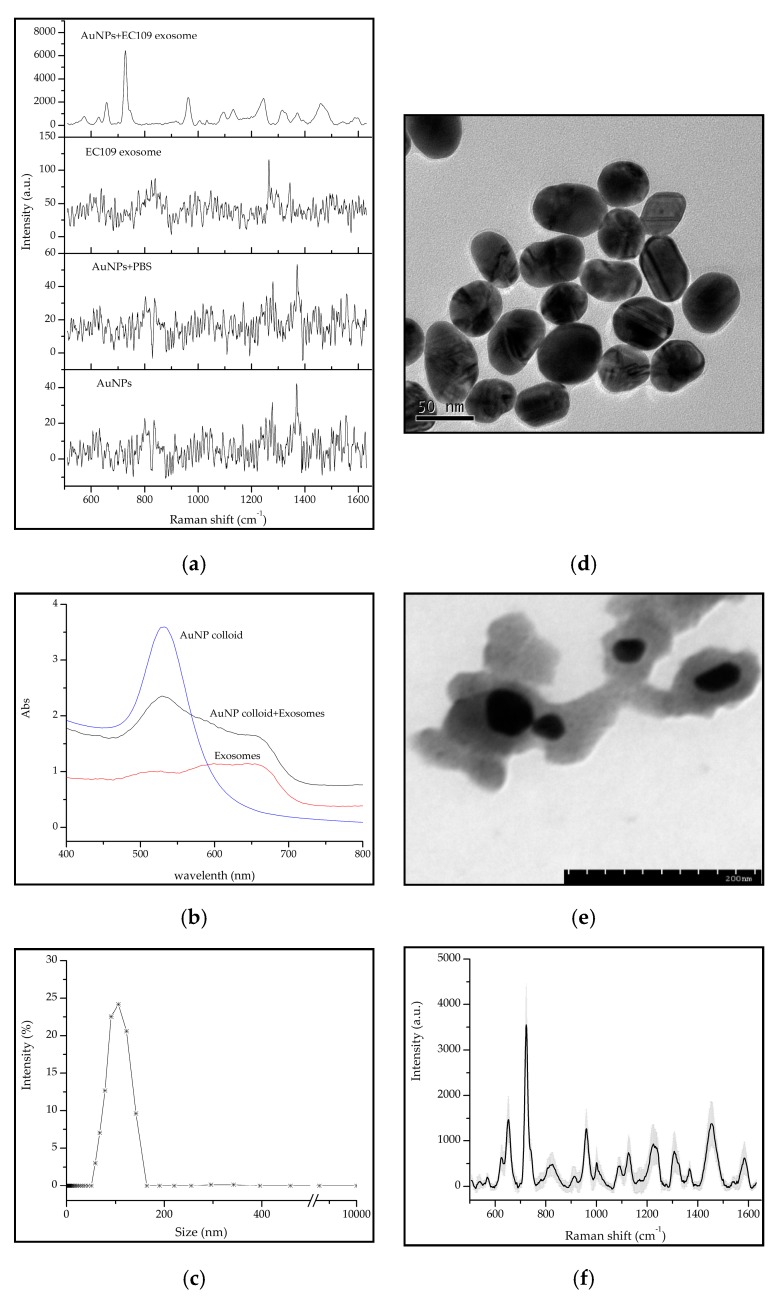
The Raman signals of exosomes were enhanced by Au nanoparticles. (**a**) The SERS and non-SERS spectra of exosomes; (**b**) Plasmon resonance absorption of Au colloid and exosomes; (**c**) The particle size distribution of Au colloid by DLS; (**d**) TEM of AuNPs; (**e**) TEM of gold nanoparticles and exosomes complex; (**f**) Mean SERS spectra (black line) and the standard deviation (gray area) of EC109 cell-derived exosome.

The normal Raman signal of exosomes suspended in PBS is rarely detected because the Raman scattering of small exosomes is an extremely inefficient process. Increasing the laser power and extending the exposure time can enhance the faint Raman signals, but since the laser power is above 10 mW, it is easy to burn the biological specimens [20]. This weakness can be overcome with application of the enhanced substrate, Au nanoparticle colloid. In Figure 2a, the SERS signal of the exosomes were enhanced as coupling with AuNPs, while under non-SERS conditions, it was immersed in the background noise. The Au nanoparticle colloid used in this study showed maximum absorption at 532 nm approximately (Figure 2b). The diameter of the gold particles was changed from 20 to 100 nm, which was consistent with the vesicular size of exosomes (Figure 1b, Figure 2c). In the TEM vision, the gold nanoparticles were nearly spherical with good dispersion (Figure 2d). As the exosomes were added into the colloid, the color of the colloid quickly turned from a red wine color into a violet color, and the Plasma absorption peak was red-shifted and the half-peak width was widened (Figure 2b). This indicates that the gold nanoparticles agglomerated and the particle size increased as combining with exosomes. Although we are not clear about the interaction between gold nanoparticles and exosomes, but from the TEM images in Figure 2e, we can infer that they may be contacted by the hydrogen bonding. Exosomes are limited by a thin lipid bilayer. The gossamer exosomes might bring surficial molecules to neighboring region of AuNPs where they could form hydrogen bonds. Due to the negative charges on the surfaces of the synthesized AuNPs, it is like to provide additional multi-electron orbitals, which can enhance the polarizability of the electron cloud in exosomes, thus enhancing the Raman intensities and improving the detection sensitivity. Nevertheless, it still requires further exploration of the type of interaction between gold nanoparticles and exosomes. Stremersch et al. reported that the enhanced Raman signals arose from the ‘hot spots’ which formed by the aggregation of gold nanoparticles and vesicles [12]. The intrinsic enhancement effect of AuNPs is proved to be related to particle size and the measured object [21]. The size similarity of Au particles and exosomes may play an important role in contributing to the SERS phenomenon, according to the physical enhancement mechanism. The enhanced Raman signals can be collected in 20 s and show high stability and reproducibility. The average standard deviations of SERS spectra did not exceed 10%, which was calculated by collecting 35 spectra in three independent experiments (Figure 2f). This indicates that the synthetic substrate has high SERS activity and therefore provides high sensitivity during rapid detection.

### 2.3. The Raman Phenotypes of Exosomes Derived from Different Types of Cancer Cells

**Figure 3:**
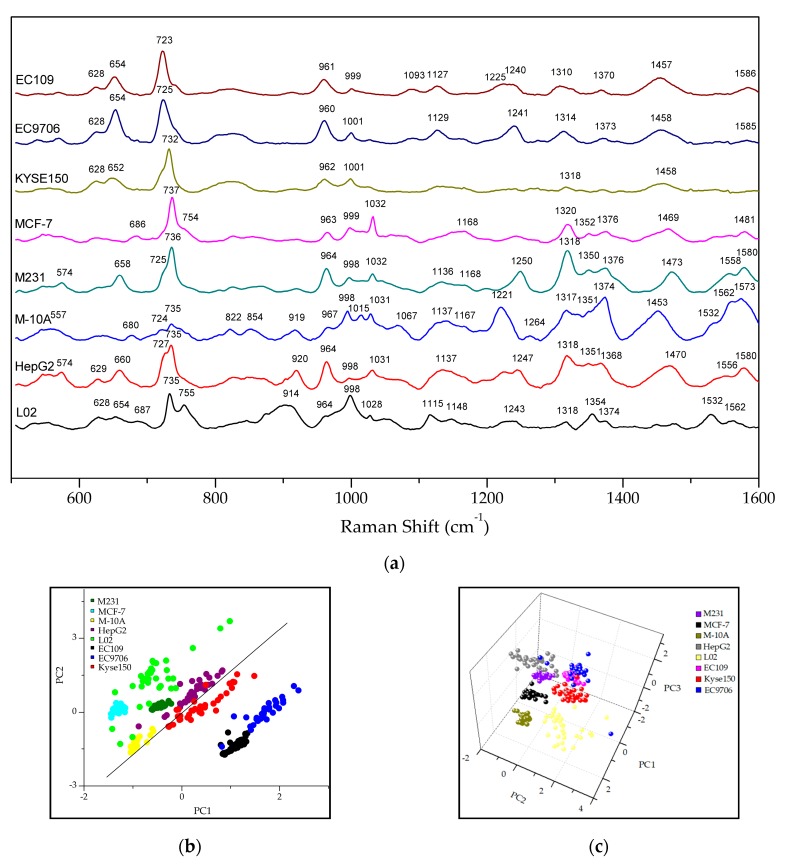
The Raman phenotypes of exosomes derived from eight different cell types. (**a**) Exo-SERS acquired from EC109, EC9706, Kyse150, MCF-7, M231, M-10A, HepG2 and L02 cells; (**b**) The two-dimensional scatter plot of PC1 (48.98%) and PC2 (23.49%) showed that the esophageal cancer cell-derived exosomes were absolutely distinguished from other cell derived exosomes by selecting the 600–760 cm^−1^ region; (**c**) Three-dimensional scatter plot of PC1 (50.03%), PC2 (12.24%) and PC3 (8.12%) showed that the breast cell-derived exosomes were absolutely distinguished from other cell derived exosomes by selecting the 940–1100 cm^−1^ region.

Figure 3a shows the average SERS spectra of exosomes derived from eight different cell line sources. Because exosomes contain the same overall structure and macromolecules (lipids, proteins, nucleic acids and so on), almost all of the Exo-SERS spectra showed common Raman peaks near 628, 730, 963, 999, 1127, 1318, 1370 and 1453 cm^−1^, which corresponding to deformation vibration of adenine ring [22], adenine vibration [22,23], adenine C-N deformed vibration [24], symmetric respiratory vibration of phenylalanine [23], C-C skeletal stretching, amide III vibration [12,22], CH deformation and CH_3_CH_2_ wagging (e.g., nucleic acids, collagen) [25], nucleotide (adenine, guanine, thymine) [24], and CH_2_CH_3_ deformation vibration, respectively [22]. However, different exosomes exhibited their own spectrum profiles, the characteristic Raman peaks and the unique relative peak intensities in the fingerprint region from 500 cm^−1^ to 1600 cm^−1^, which is the so-called distinctive exosomal SERS phenotype (abbr. Exo-SERS phenotype). From the Exo-SERS profiles, esophageal cancer cell EC109-, EC9706- and Kyse150-derived exosomes showed a higher Raman peak intensity at lower vibrational region of 700–750 cm^−1^ and a relatively lower peak intensity from 1200 cm^−1^ to 1600 cm^−1^ (Figure 3a). In the region of 600–760 cm^−1^, it showed three specific Raman peaks at 628, 654, and 723–732 cm^−1^; the highest peak was near 725 cm^−1^ which correspond to adenine respiratory vibration and C-N symmetric stretching band (phospholipid) [22,23]. It was worth pointing out that the liver cells showed up two intense Raman peaks at 720–760 cm^−1^, i.e., 727 and 735 cm^−1^ in HepG2-exosomes, 735 and 755 cm^−1^ in L02-exosomes. The vibrations at this region were mainly assigned to the nucleotide peak stretching. While in other Exo-SERS, it was only showed up one main Raman peak, which was 735 cm^−1^ in mammary exosomes, and near 725 cm^−1^ in oesphago-exosomes. Table 1 lists the characteristic Raman peaks of the eight exosomes and their assignments in detail.

When selecting 600–760 cm^−1^ to process the PC-LDA, all of the samples precisely fell into two categories, one group was the esophageal cancer cell-derived exosomes, and another was the remaining cell-derived exosomes (Figure 3b). The two-dimensional scatter plot of PC1 (48.98%) and PC2 (23.41%) showed that esophageal cancer cell-derived exosomes were absolutely distinguished from other cell-derived exosomes. In addition, a correct classification rate of 97.1% was reached when using this model to differentiate the substyles of eight exosomes (Table 2). This result indicated that the Exo-SERS phenotype in the region of 600–760 cm^−1^ was a common feature for distinguishing the esophageal cancer cells from other cell types.

The breast cancer cell M231- and MCF7-derived exosomes showed similar Raman phenotypes at the 940–1100 cm^−1^ region. This showed three specific Raman peaks at 963–967, 998 and 1032 cm^−1^, which were assigned to the C-N deformed vibration [24], symmetric respiratory vibration of phenylalanine [23], CH_2_CH_3_ bending (e.g., phospholipid) and C-C vibration (e.g., polysaccharide) [25]. Aside from the above characteristic peaks, normal breast epithelial cell MCF-10A-derived exosomes exhibited a specific peak at 1015 cm^−1^ (C–O vibration in DNA/RNA, C–C vibration) [24], while EC109-, EC9706- and Kyse150-derived exosomes had only two typical Raman peaks at about 961 cm^−1^ and 1000 cm^−1^. By applying the Raman data from 940 cm^−1^ to 1100 cm^−1^ to process the PC-LDA, the results indicated that the breast cell-derived exosomes could be correctly distinguished from other exosomes (Figure 3c). The accuracy rate was 90.6% (Table 3). Based on the PCA plots in Figure 3b,c, the more centralized the different color points representing different sample sources, the higher the similarity of SERS phenotypes of exosomes. This indicated that combining the Exo-SERS phenotypes with the PC-LDA results provided a reliable, sensitive and rapid method to non-destructively discriminate the multiple cancer types.

**Figure 4:**
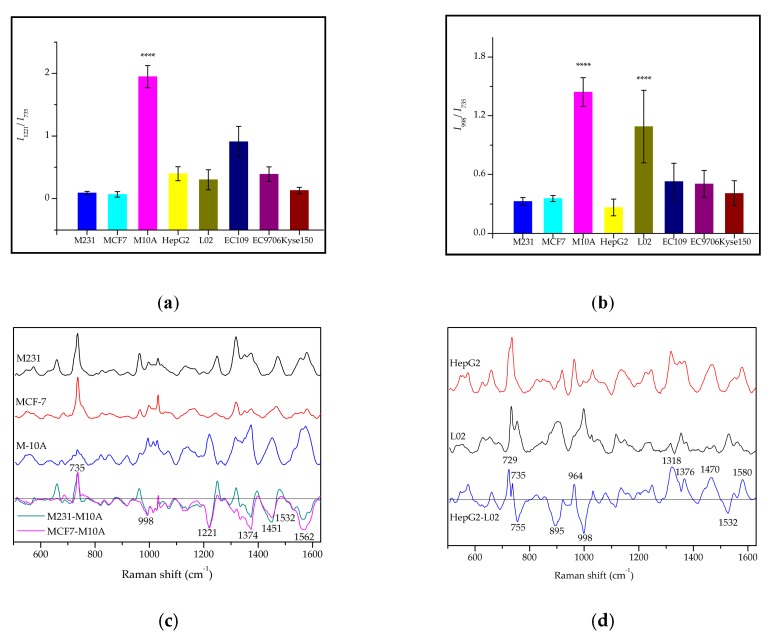
The differences between exosomal and cancerous SERS phenotypes. The relative Raman peak intensity ratio of eight different exosomes at *I_1221_*/*I*_735_ (**a**), and *I*_998_/*I*_735_ (**b**); (**c**) The differential spectra between breast cancer cell-derived exosomes and normal breast cell-derived exosomes; (**d**) The differential spectra between liver cancer cell-derived exosomes and normal liver cell-derived exosomes. Note: ****: *p* < 0.0001.

To further highlight the difference between normal cell-derived exosomes and cancerous exosomes, peak intensity ratio, and differential spectrogram were analyzed. It can clearly be seen that the Raman peaks at 1221 cm^−1^ and 998 cm^−1^ were sizable in the M10A-exosome, while the peak intensity of 735 cm^−1^ was indistinct (Figure 3a). Additionally, the peak intensity ratio of *I*_1221_/*I*_735_ and *I*_998_/*I*_735_ were above 1 in the M10A-exosome; in cancerous exosomes, by contrast, this is below 1 (Figure 4a,b). In normal liver cell L02-exosomes, the peak intensity ratio of *I*_998_/*I*_735_ is near to 1 (Figure 4b). This character could distinguish the noncancerous exosomes from cancerous exosomes in which the intensity of 735 cm^−1^ is significantly stronger than 998 cm^−1^. The scores of peak intensity ratio were significantly different (*p* < 0.0001). It is reported that 1221 cm^−1^, 998 cm^−1^ and 735 cm^−1^ are assigned to amide III attribution [24], phenylalanine ring breathing [23] and adenine ring breathing [22,23], respectively. It has been proved that diploid, triploid, binuclear, or multinuclear cells may appear in the process of cancer cell proliferation [33]. The disordered composition and metabolism of nucleic acids and proteins gives the cancer cells the power to proliferate indefinitely. The nucleic acid content of cancer cells is higher than that of normal cells. The increase of the nucleus-cytoplasm ratio may be the cause of the higher content of nucleic acids in cancers. The vesicles secreted from cancer cells contain abundant and versatile nucleic acids, including mRNA, microRNA, and DNA [1]. Because nucleic acids are rich in cancerous exosomes, the Raman signals of nucleic acids in cancer-related exosomes are much higher than that of normal exosomes. Some reports have shown that the metabolism of cancer cells is more active than that of normal cells, leading to the consumption of more proteins, lipids, and energy-supplying carbohydrates and causing lower aggregate in tissues and cells, resulting in lower reserves in cancer cells [33]. In this study, the Raman signals were produced by the Au nanoparticles adsorbed onto or nearby the monolayer membrane surface of the exosomes. Gold nanoparticles directly contact the proteins on exosomal surfaces, so the Raman signals of proteins are relatively stronger in normal exosomes and fainter in cancerous ones. For example, the vibration contributed to amino acid/proteins (919, 998, 1067, 1221, 1264, 1453, 1562 cm^−1^), carbohydrates (1374, 1532 cm^−1^) and lipids (1067, 1264, 1532 cm^−1^) on the membranes were stronger in M10A-exosomes than that of M231- and MCF7-exosomes. Thus it can be seen that changes in the relative amounts of these molecular species, or even the species ratios, can refer a measurable and useful amount of overall spectral variance. However, there still exist unknowns that require further study of the Raman information. For example, in normal exosome of L02 and M10A, the intensity of 1374 cm^−1^ (carbohydrate, A, T, G) was stronger than 1318 cm^−1^ (amide III); however, the cancerous exosomes were the opposite. The meaning of this result requires in-depth study.

The differential spectral analyses indicated differences between the Raman peaks collected from the normal exosomes and those from the cancerous exosomes. In normal M10A-exosomes, the Raman peak intensities at 1221, 1374, 1453 and 1573 cm^−1^ were significantly higher than that of the cancerous exosomes, while the intensity of 735 cm^−1^ was lower (Figure 4c). In Figure 4d, the Raman peaks at 729, 735, 964, 1318, 1376, 1470 and 1580 cm^−1^ were stronger in HepG2-exosomes than in L02-exosomes; while 755, 895, 998 and 1532 cm^−1^ were lower in HepG2-exosomes than in L02-exosomes. In addition, a typical characteristic peak at 1532 cm^−1^ showed up in L02-exosomes and M10A-exosomes, showing a significant difference from other cancerous exosomes. 

To summarize, relative peak intensity ratio and differential spectral analysis could be used as an assistant means of distinguishing the different subtypes of exosomes. We know that one SERS spectrum usually contains more than 1000 Raman bands, which provides rich and intrinsic information about the detected exosomes, reflecting the exosomes’ phenotypes and physiological states. Unfortunately, to determine all of the information is still unrealistic at present. More efforts should be put into interpreting the Exo-SERS data by means of applying Ramanomics in conjunction with proteomics and nucleomics, this will be investigated in a future study.

### 2.4. Discrimination of the Subtypes of Exosomes by PCA-LDA

**Figure 5:**
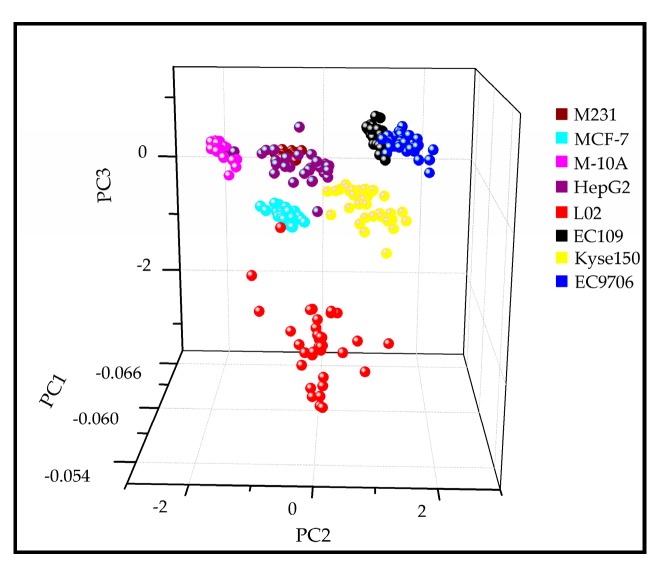
Principal component analysis of Exo-SERS data sets. The 3D scatter plot of PC1 (29.19%), PC2 (15.75%), and PC3 (11.19%) for the SERS spectra obtained from eight exosome subtypes by applying the spectral range of 500–1600 cm^−1^.

Finally, based on all of the collected Raman data, a classification model using the Raman shifts from 500–1600 cm^−1^ was established to discriminate the subtypes of different cancerous exosomes and normal exosomes, based on the PCA-LDA method. A leave-one-out cross validation was applied for the PCA-LDA result. In Table 4, the specificity of the PC-LDA model in discriminating between the subtypes of exosomes was 98.0–100%. The accuracy of the model reached 96.7%. Sensitivity was reached above 95% to distinguish the different origin-derived exosomes. Different sources of exosomes were separately concentrated in different locations in the 3D scatter plot of the PCA results, in which PC1 (29.19%), PC2 (15.75%), and PC3 (11.19%) explained 56.12% of the cumulative variance (Figure 5). 

As for EC109 and EC9706, these two cell lines are both human esophageal squamous cell carcinoma and have many common characteristics, thus they showed the same Raman phenotypes and some of the samples mixed together in the PCA model. In summary, this study established a rapid and nondestructive detection method for the analysis of cancer types based on Exo-SERS phenotypes and multivariate statistical analysis. This preliminary proof-of-concept study holds considerable promise in rapid and nondestructive detection of tumor markers by Exo-SERS technology.

## 3. Materials and Methods

### 3.1. Isolation of Exosomes in Culture Media

Human breast cancer cells MDA-MB-231 (abbr. M231) and MCF7, human hepatoma cells HepG2, human esophageal cancer cells EC109, Kyse150 and EC9706 were used to produce exosomes from different cancer types. Normal human breast cells MCF-10A (abbr. M10A) and normal human liver cells L02 were used to collect normal exosomes. All of the cells were cultured in DMEM media (HyClone, Logan, UT, USA) with 10% fetal bovine serum (abbr. FBS, Gibco, Carlsbad, CA, USA) and 1% penicillin/streptomycin (HyClone) at 37 °C under 5% CO_2_. When cell density reached approximately 60–70%, cells were washed three times with PBS (HyClone), freshly prepared DMEM containing 10% exosome-depleted FBS (Gibco) was added, and cells were cultured for 48 h. Once the cells reached 80–90% confluence, the culture media was collected for exosome isolation as depicted in Figure 1a. The supernatant was centrifuged by successive centrifugations at increasing speeds (steps 1 to 3) to eliminate dead cells and large cell debris. The collected supernatant was filtered through a 0.22 µm micron filter (Sartorius, Gottingen, Germany) and then ultra-centrifuged at 100,000× *g* at 4 °C for 80 min. After carefully removing the supernatant, the sediment was suspended in PBS and ultra-centrifuged again at 100,000× *g* for 80 min to pellet the small vesicles that correspond to exosomes [34]. The isolated exosomes were dissolved in RNase-free sterilized water and stored at −80 °C. M231, MCF7, HepG2, EC109 and M10A cell lines were obtain from the National Infrastructure of Cell Line Resource (Beijing, China). EC9706, Kyse150, and L02 cell lines were collected from BeNa Culture Collection (Shanghai, China).

### 3.2. Exosomes Identification

Exosome size distribution and morphology were tracked by dynamic light scattering (DLS, Zetasizer Nano ZS 90, Malvern Instruments Inc., Malvern, UK) and transmission electron microscope (TEM, H7650 Hitachi, Hitachi, Japan). The acquisition time of DLS was set at 10 s. The exosomes were measured via negative staining and visualization with the TEM. Then, 10 µL of sample was dripped onto a 2 mm copper mesh and precipitated for 1 min. Then, 10 µL of 3% phosphotungstic acid (pH 7.0) was added to stain for 5 min and the samples were put under an incandescent lamp to dry at room temperature. The acceleration voltage was 40 kV and the resolution was 0.14 nm. The expression of exosomal biomarkers was determined by western blotting. A primary antibody for TSG101 (BIOSS, Beijing, China), a secondary antibody to IgG-HRP (Solarbio, Beijing, China), and the control β-actin (Solarbio) was used. Protein concentration was determined by using a micro BCA protein assay kit (Thermo Scientific, Waltham, MA, USA).

### 3.3. Synthesis of AuNPs

The Au nanoparticle colloid was synthesized according to the primary work published by Wei et al. [35]. The plasmon resonance absorption of Au colloid was measured at a scanning speed of 600 nm/min in the wavelength range from 400 nm to 800 nm by the ultraviolet absorption spectroscopy (Hitachi UH4150, Hitachi Instruments Ltd., Hitachi, Japan). The Au nanoparticle size and distribution were analyzed by the method of DLS and TEM in 3.2 portion. The average particle size was calculated by using the Nano Measurer software (version 1.2).

### 3.4. SERS Detection

After mixing 10 μL of exosome solution with 10 μL of Au colloid in the PCR tubes, the mixture was immediately pipetted onto a quartz slide to collect the Raman signals. The Exo-SERS data were collected using a laser confocal micro-Raman spectrometer (inVia, Renishaw, Gloucestershire, UK). The excitation was provided by a 785 nm laser with 0.5 mW power for the exosome solution. A 50× long objective lens was applied with an exposure time of 20 s and integrated once. Approximately 35 SERS spectra from each samples were acquired at different positions. The experiments were repeated three times. Before testing, calibration was carried out through the built-in silicon wafer of the instrument to ensure that the experimental conditions were consistent.

### 3.5. Data Processing

The original data were preprocessed using Wire 4.1 software (inVia, Renishaw). The cosmic rays were removed from the original spectra and baseline correction was applied to reduce the interferences from the fluorescent background and instrument noise. The spectral regions that reflected the Raman phenotype species were cut and selected for further data analysis, including principal components analysis (PCA) and linear discriminant analysis (LDA). PCA was performed to reduce the dimensionality of the SERS data. Two or three PC scores were applied to LDA and the PC scatter plot, which displays the classification results of the different types of exosomes. A leave-one-out cross validation was used to verify the PCA-LDA results. Multivariate statistical analysis was calculated and analyzed by using SPSS software (version 19.0). One-way ANOVA was applied to compare the Raman peak intensities across different Exo-SERS. A *p*-value ≤ 0.01 was considered to be significantly different, and *p* ≤ 0.05 indicated a difference. Sensitivity and specificity is calculated according to Equation (1):

Sensitivity = TP/(TP + FP), Specificity = TN/(TN + FN)(1)

where TP is the number of dots from cancer cell-derived exosomes plotted inside the group of cancer cell-derived exosome, FP is the number of dots from normal cell-derived exosomes plotted inside the group of cancer cell-derived exosome. TN is the number of dots from normal cell-derived exosomes plotted inside the group of normal cell-derived exosomes. FN is the number of dots from cancer cell-derived exosomes plotted inside the group of normal cell-derived exosome. All of the figures were plotted by using Origin software (version 8.0) and GraphPad Prism (version 8.0.1).

## 4. Conclusions

In summary, we have reported a surface enhanced Raman scattering technology with rapid and label-free exosomal detection (Exo-SERS) for discriminating different cancer types based on specific Raman phenotypes and multivariate statistical analysis. Different types of exosomes containing different functional biomolecules resulted in specific SERS phenotypes, thus the cancer derived exosomes could be distinguished from the normal cell-derived exosomes. Furthermore, different cancer type derived exosomes varied in their SERS spectral patterns. By applying PCA, LDA, and relative Raman peak intensity analysis, the subtypes of exosomes could be accurately distinguished. The sensitivity and specificity were greater than 95% and the accuracy was 96.7% for classifying the eight exosome subtypes. This proof-of-concept study provides a rapid, label-free and non-destructive manner for detecting and discriminating between cancer types.

## Figures and Tables

**Table 1:** SERS peak positions and tentative assignments of the different cell-derived exosomes.

Raman Shift/cm^−1^								Assignments
EC109	EC9706	Kyse150	MCF7	M231	M10A	HepG2	L02	
	540		547	546	546	546	536	Cholesterol [12], C-S stretching [23]
		558	556		557	557	556	Glycogen [26]
570	570		574	574		574		C=S tensile vibration, Glycogen [26]
628	628	628	627	629	636	629	628	Deformation vibration of adenine ring, phenylalanine C-C torsional vibration [22]
654	654	652		658		660	654	Tyrosine vibration, Guanine [27]
			686		680		687	Tyrosine, phenylalanine [26]
723	725					727		Adenine respiratory vibration, nucleotides [23], C-N symmetric stretching band (phospholipid) [22]
		732	735	735	735	735	735	Adenine [22,23]
742			754		749		755	Lactic acid [14,28], DNA, nucleic acids [23], symmetric breathing of tryptophan [22]
829	826	828	828	826	822	829		Sugar–phosphate backbone vibration [27], protein [28], C-O-O vibration typical of phospholipids [29],
			855	870	854	852	847	Cholesterol, oxyproline, tryptophan, glycogen [28], C-C stretch proline ring in collagen [22]
						873	874	Tryptophan, CH_2_ deformation (e.g., protein) [25,30]
914		917	920	921	919	920	914	C=C stretching vibration, proline [24]
961	960	962	963	964	967	964	964	Adenine, C-N deformed vibration, carbohydrates [24]
999	1001	1001	999	998	998	998	998	symmetric respiratory vibration of phenylalanine [23]
					1015			C–O vibration in DNA/RNA, C–C vibration [24]
	1029		1032	1032	1031	1031	1028	CH_2_CH_3_ bending (e.g., phospholipid); C-C vibration (e.g., polysaccharide) [25]
							1055	Glycogen [28]
					1067	1071		C-C vibrations in lipid and protein [2], C–O vibration in DNA/RNA [24], collagen [26]
1093	1093							Phosphate: PO_2_^−^ vibration, C-C vibration, C-O-C vibration, glycoside link [24]
							1115	C-O ribose (e.g., nucleic acid) [30], O–P–O DNA backbone [27], C-N vibration [24]
1127	1129				1130			C-C vibrations in lipid [12,22], C-N stretching vibration in protein [22],
		1137		1136		1136		Proline [24]
			1150		1143		1148	CH vibration in protein [31], ribose-phosphate [24]
		1167	1168	1168	1167			Carotenoids [31], CH deformation in protein [30], Ribose-phosphate [24]
					1221			Amide III [24]
1225						1229	1229	Lipids, protein [28], cytosine [24]
1240	1241		1246	1250		1247	1243	Amide III [24], asymmetric phosphate stretching (e.g., nucleic acid) [25]
					1264			Amide III (e.g., protein), C=C (e.g., fatty acids) [24,25,28]
1310	1314	1318	1320	1318	1317	1318	1318	Amide III, CH deformation, CH_3_CH_2_ wagging (e.g., nucleic acids, collagen) [25], guanine [22,24],
			1352	1350	1351	1351	1354	Guanine (nucleic acid) [12], CH_2_, CH_3_ wagging in protein [24]
						1368		CH_3_ vibration (e.g., phospholipid) [12]
1370	1373	1373	1376	1376	1374		1376	Carbohydrate [12], adenine, guanine, thymine [24]
1457	1458	1458			1453		1451	CH_2_CH_3_ asymmetric and symmetric deformations in proteins, phospholipid and DNA [22,26]
			1469	1473		1470	1475	Adenine, C-N stretching, CH deformation (e.g., lipid, protein) [32]
					1532		1532	Vibration of (-C=C-) _conjugated_ [25]
				1558	1562	1556	1562	Tryptophan [12]
1586	1585		1581	1580	1573	1580		Guanine [22], adenine, purine, phenylalanine, tyrosine [24]

**Table 2:** PCA-LDA classification of eight different cell-derived exosomes by the 600–760 cm^−1^ region.

Sample	Prediction Group								Total
	EC109	EC9706	Kyse150	M231	MCF7	M-10A	HepG2	L02	
EC109	35	0	0	0	0	0	0	0	35
EC9706	1	32	0	0	0	0	0	0	33
Kyse150	0	0	36	0	0	0	0	0	36
M231	0	0	0	34	0	0	1	0	35
MCF7	0	0	0	0	35	0	0	0	35
M-10A	0	0	0	0	0	35	0	0	35
HepG2	0	0	0	2	0	1	32	0	35
L02	0	0	0	0	1	2	0	32	35

**Table 3:** PCA-LDA classification of eight different cell-derived exosomes by the 940–1100 cm^−1^ region.

Sample	Prediction Group								Total
	M231	MCF7	M10A	EC109	EC9706	Kyse150	HepG2	L02	
M231	35	0	0	0	0	0	0	0	35
MCF7	0	35	0	0	0	0	0	0	35
M10A	0	0	35	0	0	0	0	0	35
EC109	0	0	0	29	2	4	0	0	35
EC9706	0	0	0	5	26	0	2	0	33
Kyse150	1	0	0	2	4	29	0	0	36
HepG2	1	0	0	0	0	2	32	0	35
L02	1	0	0	0	1	0	2	31	35

**Table 4:** PCA-LDA classification of subtypes of exosomes derived from different cancer sources.

Sample	Prediction Group								Total	Sensitivity (%)	Specificity (%)
	M231	MCF7	M10A	HepG2	L02	EC109	Kyse150	EC9706			
M231	35	0	0	0	0	0	0	0	35	100	98
MCF7	0	35	0	0	0	0	0	0	35	100	99.6
M10A	0	0	35	0	0	0	0	0	35	100	100
HepG2	5	0	0	29	0	0	1	0	35	82.9	100
L02	0	1	0	0	34	0	0	0	35	97.1	100
EC109	0	0	0	0	0	35	0	0	35	100	99.2
Kyse150	0	0	0	0	0	0	36	0	36	100	99.6
EC9706	0	0	0	0	0	2	0	31	33	93.9	100
